# A case study of phantosmia cured by antibiotic treatment of an intranasal *Pseudomonas stutzeri* infection

**DOI:** 10.1016/j.bjorl.2021.08.006

**Published:** 2021-10-17

**Authors:** Adam G. Evans, Laurie A. Temiz, Suleman J. Bangash

**Affiliations:** a Meharry Medical College, Nashville, TN, USA; b MOHS Surgery and Dermatology Center, Elgin, IL, USA

## Introduction

Phantosmia, also referred to as a phantom smell or olfactory hallucination, is an olfactory phenomenon in which the patient perceives a smell that nobody else can smell. Phantosmia has been previously defined as “a sensation in either one or both nostrils that cannot be masked by foods”.^1^

Described etiologies for phantosmia include intracranial tumors, radiation for intracranial tumors, stroke, head trauma, surgery to the olfactory groove, an aura of a migraine, epilepsy, schizophrenia, depression, or genetic mutation. Other patients have reported an onset of phantosmia that followed viral illnesses such as influenza or infectious mononucleosis.^1^, ^2^, ^3^, ^4^, ^5^ Individuals report variable severity and characteristics of phantosmia. Phantosmia has therefore been classified by whether the perceived odor is cacosmic or torquosmic, as well as whether the perceived odor is unilateral or bilateral.^2^

We describe a patient who presented with a history of phantosmia. This patient was investigated for a nasal infection, with a nasal culture growing the bacteria *Pseudomonas stutzeri.* This patient’s phantosmia was cured by treating the nasal infection. This case report represents the first report of phantosmia due to a bacterial infection.

## Case report

The applicable institutional and governmental regulations were followed with regard to ethical human subject research, and the patient provided written consent for this study.

### Presentation

An 82-year old male with past medical history of chronic rhinitis presented to our clinic with a complaint of a phantom smell that had been present for years. This smell was worse at night, often awakening the patient from sleep, and had an ammonia-like quality. The patient reported that head movement, whether while seated or by moving to a different room could momentarily alleviate the phantom smell. He had also adopted a routine of daily Neti pot cleansing of the nares as a method of self-treatment. The patient reported that his quality of life was significantly affected by the strength, unpleasantness, and frequency of the odor. He admitted that the smell had prompted thoughts of whether life could be enjoyable and worth living while the smell persisted.

**Figure 1:**
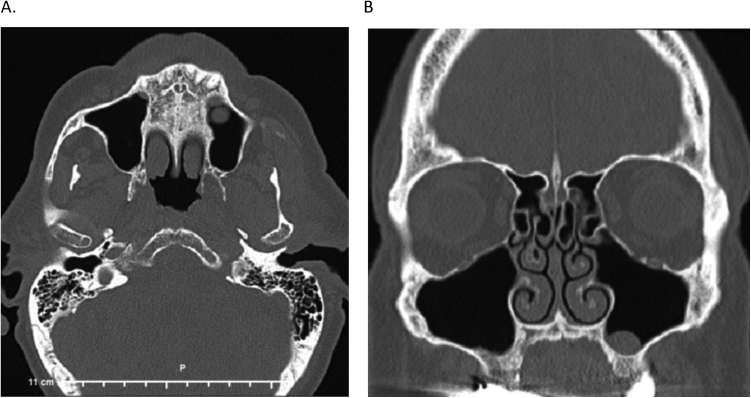
Computed tomography scan of the sinus. Axial view (A) and coronal view (B) of CT sinus scan suggest mild paranasal sinus inflammatory disease and a hyperdense structure within the left maxillary sinus that is likely a small mucous retention cyst or polyp.

He had sought help from many medical providers across several states including general practitioners, otolaryngologists, neurologists, and oral surgeons. A nasal endoscopy and CT head showed no acute pathology, however, a CT sinus showed mild paranasal sinus inflammation and a small mucous retention cyst or polyp within the left maxillary sinus (Fig. 1). Additionally, a tooth extraction in which antibiotics had been post-operatively prescribed resulted in a brief improvement of the phantom smell.

On examination, the nasal orifice was patent and without erythema or lesions. Based on the patient’s history, we obtained an aerobic culture of an area approximately 4 cm inside the external nares to rule out an intranasal infection.

### Treatment course

A presumptive treatment of mupirocin ointment was begun, and the patient was advised to discontinue Neti pot use. The culture returned, shown in Table 1, showing light growth of *P. stutzeri*, and a treatment of 2 weeks of trimethoprim-sulfamethoxazole and over-the-counter guaifenesin was initiated.

**Table 1:** Antibiotic susceptibility results of intranasally cultured *Pseudomonas stutzeri*. Intranasal culture showed light growth of *Pseudomonas stutzeri* with broad susceptibilities to antibiotics, and guided medical care to include ciprofloxacin and gentamicin.

Antibiotic	INT	MIC
Amikacin	S	<16
Cefepime	S	<4
Ceftazidime	S	<1
Ciprofloxacin	S	<1
Gentamicin	S	<2
Imipenem	S	<1
Levofloxacin	S	<2
Meropenam	S	<1
Tobramycin	S	<4

At one-week follow-up the patient reported that the smell had significantly worsened, especially at night. Mupirocin, as a pseudomonal-derived antibiotic, was discontinued due to the possibility that it was worsening the infection of *P. stutzeri*. At two-week follow-up, when the Bactrim treatment was completed, the smell was subjectively improved and had changed in quality from ammonia-like to being a sweet, but tart smell. A repeat culture was done which returned as normal skin flora.

The patient followed up at 1-year and reported that the smell would occur on-and-off. To quantify the severity of the phantosmia, a verbal numeric scale from 1 to 10 was utilized. He reported that the severity had decreased and now ranged around 1–4. His quality of life had improved, and sleep disruption had decreased. Because the patient showed improvement from the initial course, but still had significant symptoms, treatment was re-started with 5 days of ciprofloxacin and topical gentamicin to be applied daily only to the distal tip of the nares. At 2-year and 3-year follow-up the patient reported complete resolution of the phantom smell. At 5-year follow-up, the patient rated the smell as a 0 out of 10 in severity on a verbal numeric scale but reported having the gentamicin ointment refilled by his primary care provider due to a mild recurrence. Daily application of the gentamicin resolved and prevented further recurrence of the smell.

## Discussion

This case report provides the first report of phantosmia that is treatable with antibiotics. The evidence for a bacterial etiology is strengthened by the presence of the biofilm-creating *P. stutzeri* bacteria on intranasal culture, as well as the symptoms worsening initially when an empiric pseudomonal-derived antibiotic treatment was initiated.

While olfactory dysfunction in the elderly is common, severe dysfunction that warrants treatment is infrequently described. Leopold et al. described in 2002 surgical excision of the olfactory epithelium as a successful method of treating phantosmia that had been present for over 4-years.^1^ Leopold and Hornung later described in 2012 that topical cocaine use is also an effective, yet temporary treatment in all 6 of their patients.^6^ Both of these studies had resolution of phantosmia after an insult to the olfactory receptor-olfactory nerve area, suggesting that phantosmia often has an extracranial pathophysiology. Therefore, we propose that a *P. stutzeri* infection near the olfactory area was the cause of this patient’s phantosmia.

Alternatively, it remains likely that other patients may have an intracranial etiology for phantosmia. Several studies have reported phantosmia with concurrent migraine, headache disorders, or epilepsy.^3^, ^4^ Moreover, Majumdar et al. described in a 2003 case study of 2 patients that anti-epileptic medications can treat phantosmia, and Coleman et al. had similar success in using topiramate, verapamil, nortriptyline, or gabapentin.^4^, ^7^ However, the effectiveness of medical therapy alone does not confirm intracranial or extracranial etiology. A retrospective review by Morrissey et al. in 2015 described success in treating phantosmia of all 5 patients of their cohort. Following a complete otorhinologic history and examination, brain MRI, and brain CT to rule out any other pathology, they trialed haloperidol for 3-months, which was successful for 2-patients. The other 3-patients with phantosmia refractory to medical management were cured by surgical de-epithelization and cutting of the olfactory nerves.^8^ Additionally, Morrissey et al. describe the characteristics of phantosmia that is more apt to respond to surgery, which they classify as a peripheral phantosmia. A peripheral phantosmia can be triggered by another smell but is not always present; may be temporarily ameliorated (*i.e.*, nasal douche, crying, local anesthetics to the olfactory region); can be often localized to one side and may improve upon taping the nostril closed. The study of Morrissey et al. describes an algorithmic approach to treating peripheral phantosmia where medical management with antipsychotics were used prior to surgery for non-responders.

We propose that a bacterial etiology should be investigated prior to initiating antipsychotic medications. Our patient had light growth on nasal culture, which is consistent with biofilm-creating bacteria such as *P. stutzeri* exhibiting poor growth in cultures.^9^, ^10^ Alternatively, an empiric trial of topical and oral antibiotics may be considered, given the unknown accuracy of an intranasal culture. Additionally, we utilized a triple therapy, adding the expectorant guaifenesin in hopes of disrupting the biofilm by thinning the mucus.

We do not recommend an empiric treatment of mupirocin, which is an antibiotic derived from a Pseudomonal bacteria. We believe the mupirocin created a selective environment for the *P. stutzeri* to grow, which explains the worsening of symptoms after its initiation. Ultimately, antibiotic treatment of oral trimethoprim-sulfamethoxazole, and later oral ciprofloxacin, with topical gentamicin improved and cured the patient of their symptoms.

The present study has its limitations. While CT scan indicated no acute intracranial pathology, Magnetic Resonance Imaging (MRI) was not performed and is more sensitive for detecting some intracranial pathologies. Further, as a case study, the findings need to be investigated in larger populations.

## Conclusion

This patient with phantosmia was cured following antibiotic treatment of an intranasally cultured bacteria. Future research is needed to elucidate the effectiveness of antibiotics in treating more individuals with phantosmia and should investigate the accuracy of intranasal cultures in diagnosing bacterial infections existing with a biofilm.

## Funding

This research received no specific grant from any funding agency in the public, commercial, or not-for-profit sectors.

## Ethical statement

Institutional Review Board or ethics committee approval were not obtained because the present work describes a single case and does not meet the United States Department of Health and Human Services definition of “research”. The present work adhered to the Ethical principles of the Declaration of Helsinki. The patient provided verbal and written consent for this publication.

## Conflicts of interest

The authors declare no conflicts of interest.
